# Enhancers and silencers: an integrated and simple model for their function

**DOI:** 10.1186/1756-8935-5-1

**Published:** 2012-01-09

**Authors:** Petros Kolovos, Tobias A Knoch, Frank G Grosveld, Peter R Cook, Argyris Papantonis

**Affiliations:** 1 Biophysical Genomics, Erasmus MC, Faculty Building, Dr Molewaterplein 50, NL-3015 GE Rotterdam, The Netherlands; 2 Department of Cell Biology & Genetics, Erasmus MC, Faculty Building, Dr Molewaterplein 50, NL-3015 GE Rotterdam, The Netherlands; 3 Genome Organization & Function, BioQuant & German Cancer Research Center, Im Neuenheimer Feld 267, D-69120 Heidelberg, Germany; 4 Sir William Dunn School of Pathology, University of Oxford, South Parks Road, Oxford, OX1 3RE, UK

**Keywords:** enhancer, silencer, insulator, transcription factory, hub, long-range interactions, chromosome conformation capture, three-dimensional genome architecture

## Abstract

Regulatory DNA elements such as enhancers, silencers and insulators are embedded in metazoan genomes, and they control gene expression during development. Although they fulfil different roles, they share specific properties. Herein we discuss some examples and a parsimonious model for their function is proposed. All are transcription units that tether their target promoters close to, or distant from, transcriptional hot spots (or 'factories').

## Introduction

The complex linear organisation [1] of many metazoan genomes encodes regulatory sequences that can be categorised into two major groups: enhancers and silencers. Enhancers are short motifs that contain binding sites for transcription factors; they activate their target genes without regard to orientation and often over great separations in *cis *or in *trans *[2]. Silencers suppress gene expression [3] and/or confine it within specific chromatin boundaries (and thus are also called 'insulators') [4]. The interplay between these contrasting regulatory elements, their target promoters and epigenetic modifications at all levels of three-dimensional organisation (that is, nucleosomes, chromatin fibres, loops, rosettes, chromosomes and chromosome location) [5-9] fine-tune expression during development and differentiation. However, the mechanisms involved in this interplay remain elusive, although some can be computationally predicted [10]. Although enhancers and silencers have apparently opposite effects, accumulating evidence suggests they share more properties than intuition would suggest [11]. Herein we try to reconcile their apparently disparate modes of action. We suggest they act by tethering their target promoters close to, or distant from, hot spots of nucleoplasmic transcription (known as 'transcription factories') as they produce noncoding transcripts (ncRNAs) [12-15].

### Enhancers

Enhancers were characterised almost 30 years ago [16], but their functional definitions vary because of their flexibility of action (whether in *cis *or in *trans*) [17,18], position (relative orientation and/or distance) and genomic location (in gene deserts, introns and/or untranslated regions) [2]. Although sequence conservation between species can, in some cases, be an efficient predictor of enhancer identity, there are examples where genes with identical expression patterns in different species rely on enhancers that bear no similarities [19]. Within a single genome, however, sensitivity to DNase I and characteristic modifications of histone tails provide a more reliable means of identification. They typically occupy approximately 200 bp of 'open' chromatin (making them DNase-sensitive) [20], are flanked by regions rich in mono- and/or dimethylated lysine 4 of histone H3 (H3K4me1/H3K4me2) and acetylated lysine 27 of histone H3 (H3K27ac) and, generally, bind p300 [21]. Attempts have been made to classify enhancers into subclasses that are differentially used during development. Comparison between mouse embryonic stem (ES) cells, their differentiated derivatives and terminally differentiated murine cells allow distinctions between 'active', 'intermediate' and 'poised' enhancers (here additional marks are used, for example, H3K27me3 or H3K36me3) [21]. These accessible DNA stretches are often bound (and can thus be identified) by acetyltransferase p300, Mediator subunits, chromodomain helicase DNA binding protein 7, cohesin and/or CCCTC-binding factor (CTCF) [21,22]. Most importantly, canonical enhancers are characterised by the presence of bound RNA polymerase II (RNAPII) [23,24].

The first and most studied example of gene regulation by an enhancer is provided by the β-globin locus; here, the locus control region (LCR) is located 40 to 60 kb upstream from the promoter it regulates. The two interact when the chromatin fibre forms new, or rearranges preexisting, loops [17,25]. All other *cis*-regulatory elements in this locus are also in close proximity, where they form an 'active chromatin hub' [12,26]. An active chromatin hub, as defined in the β-globin locus paradigm, arises from the three-dimensional clustering of DNA-hypersensitive sites, depends on specific DNA-protein interactions and brings together all essential components for transcriptional activation [17]. Similarly, in a comprehensive study of the immunoglobulin heavy-chain locus [6] (and many other loci), the multitude of preexisting loops and connecting regulatory elements are rearranged to form new ones that interact upon activation. Obviously, most of these conformations (and in fact most seen using chromosome conformation capture (3C)) concern a population of cells and will not be refined until single-cell 3C is developed and implemented.

Enhancers are transcribed into RNAs (eRNAs) that do not encode proteins, run the length of the enhancer sequence and appear to stabilise enhancer-promoter interactions [11,24,27-29]. eRNAs derived from elements upstream of the *Arc *promoter depend on the activity of that promoter, as removing the promoter abolishes eRNA production [28]. β-globin-associated ncRNAs are still produced in the absence of the β-globin promoter [28,30,31]. However, the rate at which eRNAs are turned over, the exact mechanism by which they function and their abundance (relative to the mRNAs they regulate) all remain to be determined.

An additional class of ncRNAs longer than 200 nucleotides (long intergenic ncRNAs (lincRNAs)) were found in a survey of human transcripts, and some exhibited enhancer function [27]. In different human cells, more than 3,000 lincRNAs have now been identified [32,33]. Some seem essential for the activation of the thymidine kinase promoter, as well as for the expression of neighbouring protein-coding genes (although not all act as *bona fide *enhancers) [34]. For example, *HOTTIP *(a lincRNA transcribed from the 5' end of the *HOXA *locus) coordinates the activation of several *HOXA *genes; chromatin looping brings *HOTTIP *close to its targets, and this drives H3K4 trimethylation and transcription [35].

### Silencers

At the opposite functional extreme lie silencers. They prevent gene expression during differentiation and progression through the cell cycle [36]. This again correlates with RNA production (in some cases, through the generation of RNA duplexes that underlie the methylation of DNA at the promoter [37,38]).

Accumulating evidence supports a broad and general role of both long and short RNA molecules in transcriptional inhibition. Antigene RNAs (agRNAs) are small RNAs that target promoters and downstream regions [37]. The expression of genes encoding progesterone, low-density lipoprotein, the androgen receptor, cyclooxygenase-2, the major vault protein and huntingtin is inhibited by agRNAs [37,39]. Similarly, miRNAs, which are 20 to 22 nucleotides long, regulate gene expression post-transcriptionally [40], and they may also act at the level of transcriptional initiation or elongation. This is now supported by deep sequencing of nuclear and cytoplasmic small RNA libraries, where the majority of mature miRNAs localise in the nucleus (and not only in the cytoplasm) [41]. For instance, introduction of miRNA mimics that target the progesterone gene promoter decreases RNAPII occupancy. It also increases H3K9me2 levels in an Argonaute 2 (Ago-2)-dependent manner and leads to gene silencing [42]. Note that mature miRNAs in the nucleus can also act as 'enhancers' [43].

Polycomb complexes PRC1 and PRC2 rely on noncoding transcripts from silencing elements for recruitment to target sites. A range of examples are available: for instance, repression in *cis *in CD4^+ ^T-cells and ES cells (where PRC2-catalysed H3K27 trimethylation recruits PRC1 to prevent chromatin remodelling of targeted loci [44]) and the PRC2-*HOTAIR *interaction (where transcripts produced from the *XOXC *locus establish repression of *XOXD *[33]). In human breast cancer cells, overexpression of *HOTAIR *results in the promiscuous association of PRC2 with more than 850 targets, which are in turn silenced [45]. Furthermore, in the well-studied cascade of X chromosome inactivation, the ncRNA *Xist *binds PRC2, which in turn drives H3K27 trimethylation [46,47] and propagation of PRC1's binding to multiple sites along the silenced allele [48]. Here the three-dimensional conformation is also critical for efficient silencing and results in chromatin compaction and/or rearrangement [49]. Such equilibria may, however, be shifted by the eviction of Polycomb proteins to restore an active state [47].

### Insulators

Functionally autonomous domains are strung along the chromatin fibre, and these need to be insulated from their neighbours to prevent the action of irrelevant enhancers and silencers. Insulator or boundary elements perform this task. These can be further categorised as enhancer blockers (when the insulator is located between a promoter and a cognate enhancer) and barriers (when located between a promoter and a silencer) [50]. Mutating or deleting insulators alters the pattern of gene expression and leads to developmental defects [51].

It has been suggested that insulators evolved from a class of promoters binding a specific subset of transcription factors that drive chromatin remodelling and long-range interactions [11]. Many are marked by DNase I hypersensitivity [52] and/or the presence of bound RNAPII. Specifically, in the *Drosophila Hox *gene cluster, stalled polymerases, in conjunction with elongation factors DISF and NELF, insulate four of eight promoters from *Hox *enhancers, and this correlates with the rearrangement and/or *de novo *formation of chromatin loops [53].

Perhaps the most abundant protein associated with insulator activity is CTCF. In the well-studied example of the *Igf2-H19 *imprinted locus, CTCF prevents activation of the maternal *Igf2 *allele by a distal enhancer. When its cognate binding site is lost, the gene is reactivated [54]. However, in this locus, CTCF is a positive regulator of the *H19 *gene [45]. Moreover, CTCF mediates enhancer-promoter, insulator-insulator and insulator-promoter interactions [11]. The insulator function of CTCF is regulated by cohesins [55,56]. Their respective binding sites coincide in various cell types, including the IL-3 and granulocyte-macrophage colony-stimulating factor loci [57], as well as the *renin*, *ETNK2 *[58], *CFTR *[50,52] and *c-Myc *genes [59].

However, the CTCF-cohesin duplet is characteristic of only one type of insulator or boundary. In a comprehensive mapping of such *Drosophila *elements, additional factors, such as boundary element associated factor, GAGA and CP190, were used to identify and classify domain boundaries [60]. Again, DNase I hypersensitivity characterises many of these elements, and examples exist where their function is Ago-2-dependent (and so transcription-dependent, but RNAi-independent) [61].

### A model

**Figure 1:**
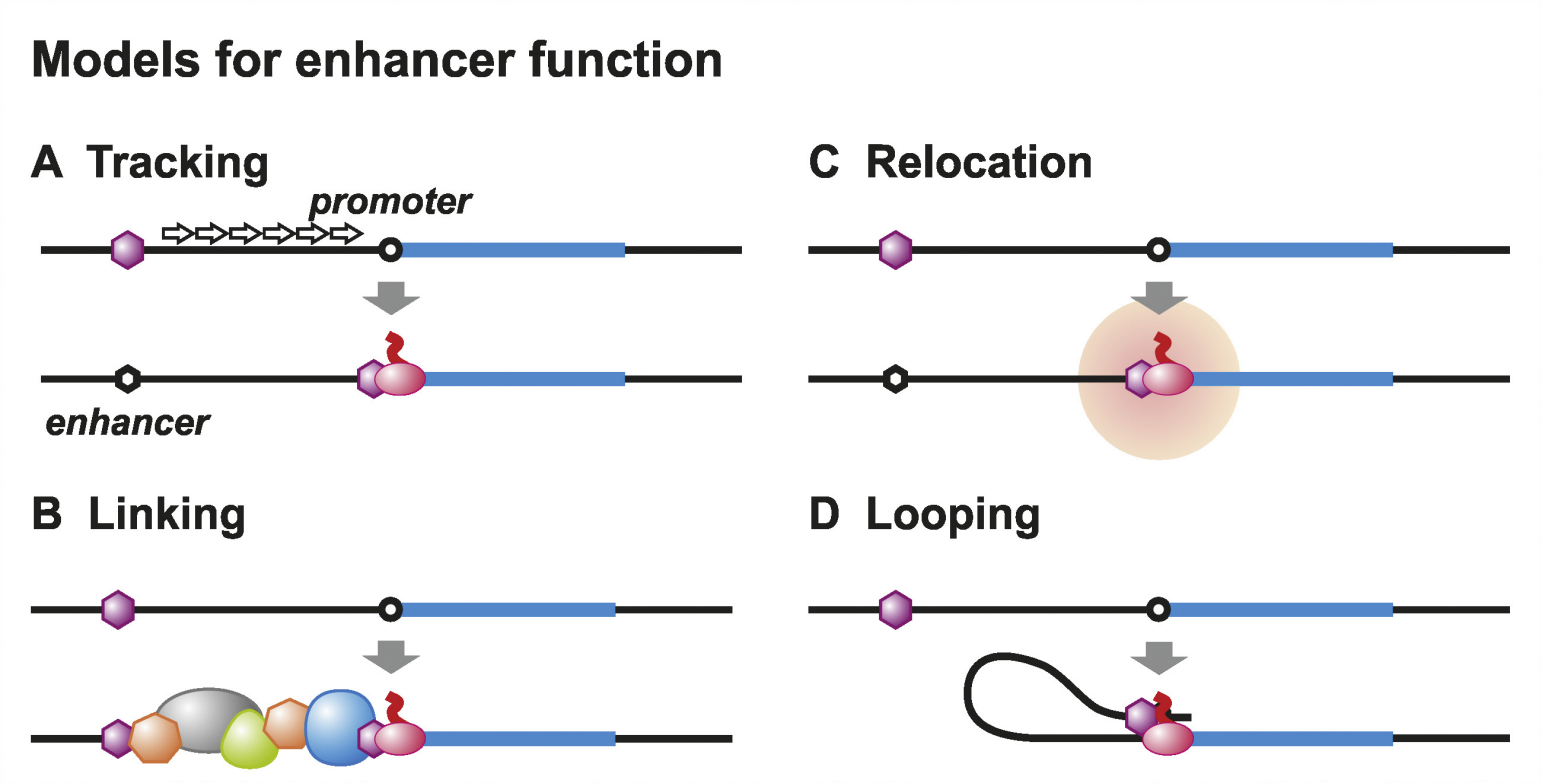
**Existing models for the function of enhancers**. The four existing models describing gene regulation by enhancers are depicted. **(A) **The tracking model, where a transcription factor (purple hexagon) loads onto the enhancer and tracks along the chromatin fibre towards the promoter, where it stimulates transcription by association with the polymerase (pink oval). **(B) **The linking model, where the loaded transcription factor drives polymerization of proteins in the direction of the promoter. **(C) **The relocation model, where a gene relocates to nuclear subcompartments (pink halo) favouring enhancer-promoter interactions, and so transcription. **(D) **The looping model, where the enhancer comes into proximity with the relevant promoter due to protein-protein interactions. This loops out the intervening chromatin and triggers transcriptional activation.

The following four models have been proposed to describe gene regulation by enhancers (Figure 1). (1) According to the tracking model, a protein loads onto the enhancer and tracks along the chromatin fibre towards the promoter, where it stimulates transcription [62]. (2) The linking model is similar, but here the loaded protein drives polymerisation of proteins in the direction of the promoter [63]. (3) In the relocation model, a given gene relocates to compartments in the nucleus where enhancer-promoter interactions (and so transcription) are favoured [64,65]. (4) The looping model (which shares features with the relocation model) predicts a direct contact between an enhancer and a relevant promoter that loops out the intervening DNA [12,65,66] and thus is closely linked to the three-dimensional genome architecture [1,7,65]. Next, activators bound to the enhancer interact with the mediator complex, which recruits RNAPII and general transcription factors to the promoter [34,67]. This last model is now favoured, as it readily explains enhancer-promoter interactions in *trans *[18,68] and is supported by a wealth of experimental data derived from 3C [69] and modelling [1,6-10,15].

Similarly, among the three major models proposed for insulator function (roadblock, sink/decoy and topological loop models), the topological loop model is best supported by experimental data: Rearrangement and/or *de novo *formation of appropriately oriented loops efficiently insulate promoters from enhancer elements [70]. Note also that recent data show how gene repression dependent on *gypsy *insulators in *Drosophila *propagates between distant loci to be repressed via the organisation of local loops [71].

Gene regulation from distal regulatory elements via local looping or broader rearrangements in three-dimensional organisation is now widely accepted. For example, we have seen that the β-globin LCR loops back to its target promoter to activate it [17] through an active chromatin hub [12,26], whereas Gata-1 represses the *Kit *gene locus via specific loop formation and exchange with Gata-2 reforms the enhancer-promoter loop and reactivates expression [72]. The *IgH *locus is another example of how this might occur, because its approximately 2.7-Mbp region is reorganised spatially during activation [6]. Similarly, various transcription factors have been implicated in forming regulatory chromatin loops, including EKLF [26]; Gata-1, Gata-2 and Gata-3 [72]; CTCF [73,74]; Ldb1 [75]; and cohesin [56,76]. Knocking them out or down results in loss of looping and changes in transcriptional state [26,77,78].

On a broader scale, the genome is organised nonrandomly in three-dimensional space [1,6-10,15] as a result of a variety of chromatin loops and rosettes [15,64,79], and the idea that transcription is also architecturally organised is gradually gaining ground [13-15]. It has been proposed that the transcription of protein-coding genes occurs in nucleoplasmic hot spots (that is, transcription factories) where a high local concentration of the required molecular machinery renders the whole process more efficient [14,15]. By definition, these harbour at least two RNA polymerases, each transcribing a different template. The β-globin active chromatin hub can be classified as a transcription factory, as it contains at least two polymerases: one transcribing the enhancer and another transcribing a protein-coding gene. Not only do active genes tend to colocalise in the nucleus to be transcribed [80,81], but different types of genes seem to cluster in 'specialised' transcription factories, where they are coregulated and expressed. For example, RNAPII genes are transcribed in separate factories from RNAPIIIgenes, whereas erythropoietic genes and TNFα-responsive genes are copied at sites distinct from those of constitutive and/or nonresponsive ones [75,82-88]. Although factories with different polymerising activities can now be isolated and their proteins characterised using mass spectrometry [89], the mechanism by which factories are 'marked' by specific transcription factors and the relative representation of different subtypes of factories remain undetermined.

How can these ideas be extended to explain the function of enhancers and silencers and/or insulators? As we have established, all share common features (for example, DNase I hypersensitivity, active chromatin marks and interaction with transcription factors and RNAPII); therefore, we propose that canonical regulatory elements are primarily transcription units (Table 1) and that, in order for them to be functional, they need to be transcribed (and so associated with a transcription factory). This hypothesis defines two key aspects of chromatin structure: proximity between distant DNA sequences due to looping and tethering of active genes to a factory.

**Table 1:** Examples of genes or loci associated with enhancers, silencers or insulators^a^

Gene/locus	Association	Type	Reference
IgH locus	mU element	Enhancer	[6]
β-globin locus	LCR	Enhancer	[12,17,26]
*Kit*	-114 kb	Enhancer	[72]
*Arc *promoter	eRNA	Enhancer	[28]
*HOXA *locus	*HOTTIP*	Enhancer	[35]
Prostate cancer cells	Androgen receptor	Enhancer	[20]
*Sox2*	SRR1/2	Enhancer	[90]
*HO-1*	+12.5 kb	Enhancer	[91]
Progesterone receptor	agRNA, miRNA	Silencer	[39,41]
Androgen receptor	agRNA	Silencer	[37]
*Cyclooxygenase-2*	agRNA	Silencer	[37]
LDL receptor	agRNA	Silencer	[37]
Major vault protein	agRNA	Silencer	[37]
*Huntingtin*	agRNA	Silencer	[37]
Various target genes	Polycomb	Silencer	[44]
*INK4-ARF *locus	Polycomb	Silencer	[100]
*XOXD *locus	HOTAIR	Silencer	[33]
X chromosome	Xist	Silencer	[42]
Chicken β-*globin *locus	5'HS4	Insulator	[92]
*ApoB *locus	5' end of gene	Insulator	[93]
*Drosophila HOX *locus	Stalled RNAPs	Insulator	[53]
Mouse *Igf2-H19 *locus	CTCF	Insulator	[50]
Dystrophy locus	CTCF	Insulator	[94]
*IL-3*	CTCF	Insulator	[57]
*GMCSF *locus	CTCF	Insulator	[57]
*Renin*	CTCF	Insulator	[58]
*ETNK2 *locus	CTCF	Insulator	[58]
*CFTR *locus	CTCF	Insulator	[95]
*c-Myc*	CTCF	Insulator	[55]
Chicken α-*globin *locus	CTCF	Insulator	[96]

^a^agRNA, antigene RNA; CFTR, cystic fibrosis transmembrane conductance regulator; CTCF, CCCTC-binding factor; eRNA, enhancer RNA; IgH, immunoglobulin H; LDL, low-density lipoprotein; RNAP, RNA polymerase.

Does the number of factories in a given cell suffice to accommodate all transcription units, including enhancers and/or silencers? To date, the lowest estimate of about 200 factories concerns murine primary cells and comes from RNAPII immunostaining *ex vivo *[81]. This suggests that about 80 transcription units would share a factory (assuming 16,000 active transcription units, as in HeLa cells) [86] or that a number of them are transcribed outside a factory. Other approaches in HeLa cells return a number that is an order of magnitude higher: approximately 2,000 factories, each hosting an average of 8 transcription units [97,98]. Moreover, the density and diameter of these transcriptional hot spots appear to be constant between cell types, suggesting an underlying topology accessible to transcription units in different nuclear neighbourhoods [86,99]. The difference between these numbers may be explained by a difference in sensitivity of detection [86,98]. But does most transcription occur in factories? It seems it may, as some estimates indicate that more than 95% of nascent nucleoplasmic RNA is found in factories (assessed using incorporation of various precursors in a variety of cell types) [13,97-99]. Nonetheless, these issues will probably be resolved only by imaging factories in different types of living cells.

**Figure 2:**
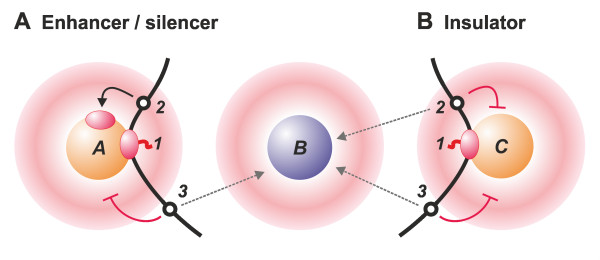
**A simple model for the function of regulatory elements**. Spheres A, B and C represent factories rich in different sets of transcription factors and associated halos indicate the probability that promoter *1*, *2 *or *3 *will collide with a factory (red indicates high probability). The low-probability zone immediately around the factory arises because the intrinsic stiffness of the chromatin fibre restricts the formation of very small loops). Curved black arrow indicates collision between promoter and factory that yields a productive initiation. Dashed grey arrows indicate the preferred site of initiation (as factory B is rich in the relevant transcription factors). Blocked red arrows indicate unproductive collisions (as the factory contains few of the relevant factors). **(A) **Enhancers and silencers. Transcription unit *1 *is being transcribed by a polymerase in factory A. This tethers unit *2 *in a 'hot zone', where it has a high probability of colliding with a polymerase in factory A (which contains high local concentrations of factors necessary for initiation by promoters *1 *and *2*). As a result, unit *1 *acts as an enhancer for unit *2*. At the same time, unit *3 *is tethered far from factory B (which is rich in the factors required for its initiation). Here unit *1 *acts as a silencer of unit *3*. **(B) **Insulator. At a different stage in development, a different constellation of transcription factors are expressed. Chromatin domains containing units *2 *and *3 *are separated by unit *1 *(now transcribed in factory C, which contains low concentrations of the factors required by units *2 *and *3*), so they rarely bind to factory A and interact. Here unit *1 *acts as an insulator or barrier.

Now consider that an enhancer (transcription unit 1) (Figure 2A) tethers its target promoter (in unit *2*) close to factory or hub A that contains the necessary machinery. As a result, the target promoter 2 will diffuse through the nucleoplasm and frequently collide with a polymerase in factory A to initiate transcription. Although promoter 3 is also tethered close to the same factory, it will initiate rarely (because factory A lacks the necessary transcription factors required by this particular promoter). Although promoter 3 can initiate in factory B (which contains high concentrations of the relevant factors), it will do so rarely, simply because it is tethered close to factory A and far from B. Next, transcription unit *1* acts as an enhancer of unit *2* and as a silencer of unit *3*. The addition of histone modifications that mark the various units as active or inactive will now reinforce the *status quo*. After that, once unit *1* has been transcribed, these marks will make it more likely that unit *1* or unit *2* will reinitiate in factory A to create a virtuous cycle. Similarly, at another developmental stage, when a different set of transcription factors are expressed (Figure 2B), unit *1* might be transcribed in factory C. It is again flanked by units *2* and *3*, but these can now be transcribed efficiently only in factory B (which is rich in the necessary factors). As units *2* and *3* cannot stably interact with each other by binding to factory C, unit *1* now acts as an insulator or barrier. As before, histone marks will reinforce this different (virtuous) cycle.

## Conclusions

The model we propose here (Figure 2) illustrates a case where nongenic transcription unit 1, in its normal genomic location, acts as an enhancer, silencer or insulator or barrier, depending on the target and developmental stage. We imagine that most regulatory motifs normally act in only one way; however, when moved out of their normal context (usually the case in the assays used to test for the action of these motifs), they will act differently, depending on the new context (which includes proximity to an appropriate factory). This model encapsulates notions of transcriptional activity, epigenetic marks and three-dimensional architecture, which, in combination, provide the context that determines promoter activity.

## Abbreviations

bp: base pair; kb: kilobase; IL: interleukin; Mbp: megabase pair; miRNA: microRNA; RNAi: RNA interference; TNF: tumour necrosis factor.

## Competing interests

The authors declare that they have no competing interests.

## Authors' contributions

All authors structured, wrote and proofread the manuscript.
